# Does Friend Support Matter? The Association between Gender Role Attitudes and School Bullying among Male Adolescents in China

**DOI:** 10.3390/children9081139

**Published:** 2022-07-29

**Authors:** Binli Chen, Xiying Wang, Yutong Gao

**Affiliations:** 1 School of Social Development and Public Policy, Beijing Normal University, Beijing 100875, China; blichen@bnu.edu.cn; 2 Institute for Education Theories, Faculty of Education, Beijing Normal University, Beijing 100875, China; 3 School of Education, University of North Carolina at Chapel Hill, Chapel Hill, NC 27599, USA; gyutong@unc.edu

**Keywords:** adolescent bullying, gender role attitudes, friend support, masculinity

## Abstract

This study investigated the association between gender role attitudes, perceived friend support, and school bullying among male adolescents from 11 schools in two cities in China. A total of 3172 Chinese adolescents between 12 and 20 years of age (48.80% girls and 51.20% boys) completed questionnaires that included measures of bullying, gender role attitudes, and perceived social support. In terms of outcome measures, the Chinese version of the Illinois Bully Scale (IBS), Attitudes toward Women Scale for Adolescents (AWSA), and Multidimensional Scale of Perceived Social Support (MSPSS) were used to assess bullying perpetration, gender role attitudes, and perceived friend support, respectively. Based on masculinity theories and the stress-buffering theory, the study found that male adolescents held more traditional gender role attitudes (t = 30.78, *p* < 0.001) and reported higher prevalence of bullying behaviors (36.02%) than girls (31.20%). In addition, boys’ bullying behaviors were significantly predicted by gender role attitudes through perceived friend support. That is, male youth with more conservative gender role attitudes reported less perceived friend support (adjusted OR = 1.055; SE = 0.013), which elevated their risks of bullying perpetration (adjusted OR = 2.082; SE = 0.302). These findings have critical implications for bullying intervention and prevention through gender equity education.

## 1. Introduction

School bullying is a worldwide public concern. Bullying, defined as “repeated exposure to aggressive behavior from peers with the intent to inflict injury or discomfort,” is characterized by: (1) intentional harm; (2) recurring nature; and (3) power or strength imbalance between the perpetrator(s) and the victim [1,2,3]. Common forms of bullying include physical attacks, verbal abuse, and psychological or relational violence [1]. Globally, it is estimated that 246 million children and youth are impacted by some form of school violence and bullying every year [4]. Additional data show that approximately one in three students (32%) reported at least one incident of bullying victimization at school in the prior month [5]. Overall, an estimated prevalence of bullying involvement ranges from 29% to 46% across studies and regions [6].

The severity of school bullying is also increasingly recognized in China. Reviews of Chinese studies reported that 2% to 66% of students had experienced bullying victimization and 2% to 68% had engaged in bullying perpetration, with great heterogeneities due to study methodology (e.g., using different measurement scales, different time span, and different definitions of school bullying) [7]. In their study in seven Chinese provinces, Xue and colleagues found that approximately 17.3% of 3675 high school students reported being involved in school bullying as perpetrators [8]. Meanwhile, according to data from the Program for International Student Assessment (PISA) 2015 and 2018, 22.5% and 17.7% of 15-year-old students from four regions in China reported being victimized at least a few times per month [9,10,11,12]. Extensive research in China and abroad has pointed out the negative impact of school bullying, ranging from academic underachievement [13] to psychosocial problems, including low self-esteem, depression, anxiety, and suicidal ideation [14,15,16,17,18]. Clearly, for better prevention of the behavior, continuing research, particularly the identification of protective and risk factors, is needed.

Notably, research shows that bullying experience varies considerably across gender. First, numerous studies have reported higher rates of bullying perpetration among adolescent boys than girls. One large-scale cross-national study, Global Student Health Survey (GSHS), shows that that the global prevalence of bullying among adolescents aged 13 to 15 is 30.4% among girls and 34.8% among boys [5]. Data from another international survey, Health Behavior in School-Aged Children (HBSC), show similar findings. According to the 2017/2018 HBSC report, which analyzed results among approximately 220,000 adolescents from Europe and North America, boys aged 11, 13, and 15 in over half of the nations reported higher rates of perpetration than girls, with an average gender difference of 3% [19]. Second, it seems that boys and girls are involved in different bullying behaviors. While boys are more likely to engage in physical bullying and violence, girls are more likely to adopt verbal, relational, and psychological abuse [4,5]. In terms of victimization, evidence suggests that compared with boys, who are more likely to be physically attacked, girls are at greater risk of sexual violence victimization and exploitation [4,5]. Further, girls are more likely than boys to report combined victimization of cyberbullying and traditional school bullying, and their experience of cyberbullying is often associated with sexual abuse [5,20].

Similar gender differences are also observed in China: overall, male students are more likely to be perpetrators and more prone to victimization than their female counterparts [21]. For instance, in a large-sample national survey on school bullying, with more than 100,000 students from Grade 4 to 11 from 29 provinces in China, boys in each grade reported higher rates of perpetration and victimization than girls [22]. Another study conducted among 22 middle schools in Suzhou, Jiangsu province found that 26.4% of boys and 14.3% of girls had been involved in bullying at least once in the last semester [23]. An earlier study by our research team using the same data set as the present study also revealed that about 16.85% and 11.11% of male and female students had been involved in school fighting [24]. Based on the existing findings, the present investigation focused on male students’ school bullying behaviors and particularly predictive factors.

Previous studies indicate that bullying involvement among male students is affected by traditional gender role attitudes and gender-related stereotypes that persist in society. Gender role attitudes (or gender ideology, gender-related beliefs) are individual opinions about appropriate societal roles based on gender [25]. Traditional or rigid gender role attitudes support polarized gender attributes and traits for males (e.g., leadership, agency, aggression, bravery) and for females (e.g., childrearing, submissiveness, tenderness, kindness) and presume male superiority over females [26]. By contrast, egalitarian gender role attitudes endorse a more flexible gendered division of responsibilities and advocate gender equality. In this case, school bullying reflects underlying social norms regarding authority and expected gender roles. Dominant conceptions of manhood may lead to tolerance of boys acting out expressions of aggression, violence, sexual power, and homophobia [25]. Research has demonstrated the association between traditional gender beliefs and greater involvement in aggressive behaviors, including school bullying [27,28,29].

Further, perceived friend support, a critical aspect of social support, may be a potential mediator linking gender traditionality and male bullying behaviors. Social support is broadly defined as supportive behaviors and assistance provided by members of one’s social networks [30]. It may take different forms, including emotional (e.g., empathy, love), instrumental (e.g., money, resources), informational (e.g., advice), and appraisal support (e.g., feedback) [30]. During adolescence, a wide range of psychosocial transitions occur, including the expansion of social networks [31]. For youth, friends gradually become a more significant source of social support than family members [32]. Extant research has generally appraised friend support as a contributor to individual physical and mental well-being [33,34,35]. However, increasing evidence has shown that traditional gender role attitudes, particularly among males, are associated with the perception of less friend support, which leads to a wide range of psychosocial problems, including aggression [36].

### 1.1. Literature Review

#### 1.1.1. Gender Role Attitudes among Chinese Adolescents

In China, despite rapid economic development and the impact of globalization, patriarchal traditions derived from Confucianism continue to rule [37,38,39]. Further, in the education domain, access to gender equality knowledge is limited: comprehensive sexuality education, which incorporates themes of gender equity education, is lacking [40]. Consequently, a large proportion of Chinese youth still holds conservative gender role beliefs that essentialize stereotypical gendered behaviors and attributes [41,42,43,44,45].

While gender traditionality remains influential in younger generations, male adolescents appear to hold more conservative beliefs than females [40,42,46,47,48]. Males, privileged by patriarchal systems, are more likely to support traditional division of labor and resist egalitarian norms [25].

Such a phenomenon is also reported in both global [46] and emerging Chinese research. In a large-scale study by Zuo and colleagues [47] that recruited 5709 adolescents and emerging adults from six provinces in China, male respondents consistently reported higher endorsement of gender traditionality in all domains, including family, public, and intimate relationship domains. Particularly in family and public domains, average gender differences exceeded 10%, as considerably higher proportions of adolescent boys scored higher than females in most items. Echoing such findings was Tang and colleagues’ study, which involved 1527 youth from fifth graders to college juniors based in Changchun, a large city in northeastern China [48]. Results indicated that male youth generally held less egalitarian attitudes than females across all age-groups. Similar gender gaps have been reported in other cross-sectional studies in China [42].

Such gender gaps, as evidenced by the limited yet growing longitudinal research, may remain significant across time [40]. In a study by Sa and colleagues that evaluated the effectiveness of a comprehensive sexuality education program involving 1131 10th graders in Lanzhou, China, significant increases in gender egalitarianism were found for female youth only [40]. This suggests that gender traditionality among males is entrenched and more resistant to change.

#### 1.1.2. Traditional Gender Role Attitudes and Adolescent Male Aggression

The association between traditional gender role attitudes and adolescent aggression is through in several theoretical frameworks. One such framework is hegemonic masculinity. The term refers to the most valued masculine norm in one society, often associated with authority and power [49]. It serves to sustain male dominance over females and is central to patriarchy [49,50]. Contemporary hegemonic masculinity incorporates several themes, including dominance and violence [51]. Such themes, as global research reveals, are largely shared among patriarchal societies, and China is no exception [52,53,54,55]. One Chinese survey that investigated masculine norms and violent behaviors showed that among 1981 participants, 21.8% of female and 52.4% of male respondents agreed that “a man can defend his reputation through violence if he has to” [54]. Accordingly, boys raised in such norms learn to behave in corresponding ways through reinforcement and punishment of multiple socializing agents (e.g., family, peers, media, schools). Thus, based on this perspective, the disproportionately high perpetration of violence in males—in this case, more bullying behaviors in adolescent boys—results from the socialization to hegemonic masculinity that promotes aggression.

Much evidence exists that supports the impact of traditional gender role beliefs on intimate partner violence and sexual assault [43,52,53,54,55,56], as well as dating violence [57,58,59,60,61], homophobic name-calling [62,63,64], and fighting behaviors [63] among male adolescents. In terms of adolescent school bullying, one study was conducted among 206 high school football players in the Midwestern US, and reported their bullying behaviors in the past 2 months and traditional masculinity beliefs, along with other items [27]. Results suggested that higher endorsement of traditional masculine norms significantly predicted bullying perpetration. Similar patterns were found in two other studies, which analyzed both female and male samples. Another study employed a Croatian sample of 4072 10th and 11th graders [28]. Among both girls and boys, bullying perpetration was significantly predicted by higher scores on the masculine norm measure, indicative of more conservative masculinity attitudes. Likewise, Carrera-Fernández and colleagues, in their investigation involving 1500 Spanish adolescents, found that hostile sexism was indirectly associated with bullying behaviors through instrumental gender traits and pro-bullying attitudes among both girls and boys [29].

#### 1.1.3. Traditional Gender Role Attitudes, Perceived Friend Support, and Aggressive Behaviors

The reason that perceived friend support is chosen as a mediator between gender traditionality and bullying is twofold. First, among males, holders of traditional gender role attitudes tend to restrain emotion and refrain from seeking help. Such tendencies are postulated in theories of hegemonic masculinity and are empirically supported. According to theorists, the dominant masculine norm encourages toughness and self-reliance for males [65]. In this light, emotional expression and help-seeking are incompatible with the norm and therefore discouraged in boys’ gender socialization [66]. One who refuses to disclose his feelings and concerns may therefore perceive less peer support. The hypothesis is supported by several studies among adult males. For example, in one study that investigated masculine beliefs and health-related behaviors among 546 male college students, higher adherence to traditional masculine norms was significantly connected with tendencies to handle problems alone and refusal to admit sickness or express feelings to others [67]. Similarly, another study conducted among 396 male undergraduates found that higher scores of restricted emotionality and restricted affective behaviors among men were predictive of less social support, which contributed to greater psychological distress [68]. In terms of adolescent research, one systematic review that analyzed 20 quantitative studies on youth masculinity, internalizing behaviors, and social support found that higher endorsement of traditional masculine norms was associated with lower levels of self-reported social support [69].

Second, the lack of peer and friend support has often been associated with aggressive tendencies. The stress-buffering hypothesis conceives that social support protects one against stressful events by altering negative appraisals of the event or providing resources to tackle the problem [70]. Either way, adequate social support enables one to address negative emotions and cope with difficult situations effectively. By contrast, youth who perceive insufficient social support may fail to deal with stress and adopt problematic coping strategies, including bullying behaviors. One recent meta-analysis on social support and problem behaviors among adolescents has provided support for the hypothesis. The study, which examined 51 studies on the impact of general social support among 196,247 youth, reported that social support significantly reduced the likelihood of bullying perpetration among adolescents [33]. Studies that have looked at distinct forms of support have generally yielded similar findings. For instance, in the 2013/2014 HBSC survey, which comprised 43,667 prepubescent and pubescent participants from nine European countries, all types of social support, including friend support, were related to lower odds of violent behaviors (bullying included), although family support had the strongest protective effect [34]. Additionally, one longitudinal study that followed 880 secondary school students for 1 year found that greater friend support at baseline predicted less bullying perpetration at follow-up [35].

### 1.2. Research Questions and Research Hypotheses

This study intended to examine the association between gender role attitudes, perceived friend support, and school bullying among Chinese adolescent boys. In particular, the study extends the existing literature on the relations between gender role attitudes and bullying perpetration by addressing two questions. (1) What is the relationship between gender role attitudes and bullying perpetration among male Chinese adolescents? (2) Is perceived friend support a mediating factor between gender role attitudes and bullying perpetration?

Based on the above findings of the associations between traditional gender attitudes, perceived friend support, and bullying behaviors, two hypotheses are proposed:

**Hypothesis** **1** **(H1).** *Male adolescents who hold more traditional gender role attitudes will have higher prevalence of bullying perpetration.*

**Hypothesis** **2** **(H2).** *Male adolescents with more traditional gender role attitudes will report lower levels of perceived friend support, which is associated with a higher prevalence of bullying perpetration.*

## *2.* Materials and Methods

### 2.1. Sample and Data Collection

Data in the present study were obtained from a survey named the Study on School Life of Teenage Students in China, which was conducted by the research team in, Suqian, Jiangsu, and Chongqing in 2018. Suqian is a medium-sized city in Jiangsu province, with a population of about 4.9 million. Chongqing, a large city in western China, is home to about 30 million people. For participant recruitment, stratified sampling was employed: In the first stage, schools were stratified according to urban and rural areas and the type of school (junior high schools, senior high schools, or secondary technical schools). Eleven schools (six junior high schools, four senior high schools, and one vocational school) were selected during this stage. In the second stage, four to five classes were randomly selected in each school. In the third stage, all students in selected classes were included in the sample.

The questionnaires were distributed with the permission and assistance of school administrators and staff, and students completed informed consent forms. Students filled in the questionnaires independently during class time while the researchers stayed in the classroom, offering guidance and assistance when needed. In total, 4000 questionnaires were distributed, and 3531 questionnaires were collected, for a response rate of 88.3%. A total of 331 were excluded due to a large amount of missing data, which resulted in a final sample of 3172: 1624 male students and 1548 female students. Table 1 shows the sociodemographic characteristics of the final sample. Table 2 presents descriptive statistics of bullying perpetration, gender role attitudes, and perceived friend support among boys and girls, respectively. Regression analysis for key variables in the male sample is included in Table 3.

### 2.2. Measures

#### 2.2.1. Bullying Perpetration

In measuring adolescent bullying perpetration, the present study used the bullying subscale of the Chinese version of the Illinois Bully Scale (IBS) [71,72], which has been shown to have good reliability [24,73]. The subscale includes nine items to assess bullying behaviors in the past 30 days. Sample items include: “I upset other students for the fun of it,” “I teased other students,” and “I helped harass other students.” The response options include 0 (never), 1 (one or two times), 2 (three or four times), 3 (five or six times), and 4 (seven or more times). A higher score indicates more frequent involvement in the behavior. The internal consistency of the bullying subscale was acceptable in the current sample (Cronbach’s alpha= 0.80). In this study, bullying was coded as a dummy variable, with 1 representing “involved in bullying” and 0 representing “not involved in bullying.” Following previous studies, “three or four times” was used as the threshold value for coding whether a student was involved in bullying behavior [24]. That is, a student who had engaged in a bullying behavior at least 3–4 times in the past 30 days was coded as a bullying perpetrator. Others were coded as the non-bullying group.

#### 2.2.2. Gender Role Attitudes

The translated Chinese version of the Attitudes toward Women Scale for Adolescents (AWSA) [74] was used to measure gender role attitudes. This 12-item scale was measured on a four-point Likert scale ranging from 1 = “agree strongly” to 4 = “disagree strongly.” Sample items of the AWSA include “Swearing is worse for a girl than for a boy” and “It is more important for boys than girls to do well in school.” This study is the first to apply this scale to a Chinese sample. Based on preliminary results of exploratory and confirmatory factor analysis for the present version, this study found that the factor loadings for item 2 and item 7 were low. Therefore, these two items were excluded from the final calculation of the total gender attitude score. Overall, a higher total score on the scale indicates a more egalitarian gender role attitude. In the present study, the scale’s internal consistency was acceptable (Cronbach’s alpha = 0.70).

#### 2.2.3. Perceived Friend Support

For perceived friend support, the present study used the subscale of friends’ support of the Multidimensional Scale of Perceived Social Support (MSPSS) developed by Zimet and colleagues [75], which has been shown to be reliable in studies among Chinese adolescents [76,77]. The subscale contains 4 items: “My friends really try to help me,” “I can count on my friends when things go wrong,” “I have friends with whom I can share my joys and sorrows,” and “I can talk about my problems with my friends.” Response options range from 1 (very strongly disagree) to 7 (very strongly agree). A higher score indicated that respondents have more support from friends. The internal consistency of the subscale in the current sample was high (Cronbach’s alpha = 0.85). Based on the aggregated item scores, this study categorized friend support into high, medium, and low levels using one standard deviation above and below the mean (21.44 + 5.56).

#### 2.2.4. Control Variables

Adolescent bullying behaviors are also influenced by numerous other factors [7,78,79]. Therefore, several other variables were controlled for during data analysis. The first set of control variables was related to parental problem behaviors. Previous studies have found intergenerational transmission of violence in the family, with parents’ problem behaviors significantly increasing the likelihood of children’s bullying or aggression [78,79]. Accordingly, several items in the questionnaire were accounted for, including those regarding parental smoking and drinking history, as well as spouse and child abuse (Yes = 1, No = 0). The answers to these questions were summed as the total score for paternal and maternal problem behaviors, respectively. The second set of control variables were adolescent characteristics, including age, stage of education attended (dummy variable, “junior high school = 1” and “senior high school = 0”), household registration (urban area = 1 and rural area = 0), being an only child (No = 0, Yes = 1), and passing all exams in the past semester = 1) [80,81,82]. Finally, family socioeconomic status were also controlled for, including parents’ years of education, marital status (dummy variable, married = 1), and self-rated family economic status (dummy variable, better = 0) [81,82]. The sociodemographic and control variables of the sample are described in Table 1.

### 2.3. Data Analysis

We analyzed the data using Stata software 14.0 (StataCorp, TX, USA, 2015). We first present the sample distribution of adolescent boys’ sociodemographic characteristics and bullying scores, gender role attitudes, and perceived friend support by gender groups (see Table 1 and Table 2). Second, three regression models were used to examine the relationship between gender attitudes and bullying and whether perceived friend support mediated the relationship (Table 3). Since bullying is a dichotomous variable and friend support is an ordinal variable, this study used binary logistic regression models and an ordinal logistic regression model to predict bullying and friend support, respectively. Third, the Sobel–Goodman test was used to examine the mediating effect of perceived friend support.

## 3. Results

### 3.1. Descriptive Statistics

The mean age of adolescent boys was 14.94 years (Table 1). Of all the 1624 boys, 44.89% of the respondents were attending junior high school, and 55.11% were attending senior high school. Of the boys, 37.81% were from rural areas and 35.28% were the only child in their family, 64.96% had not passed all their exams in the last semester, and 88.49% of the respondents’ parents’ marital status was in marriage. In addition, 16.75% of the boys rated their family’s financial status as better, and 14.35% rated as worse.

As Table 2 suggests, 36.02% of adolescent boys reported bullying behavior in the past month, which was higher than the percentage in girls (31.20%). Meanwhile, boys’ gender role attitudes scores were significantly lower than girls’, indicating that boys held more traditional gender role attitudes. Additionally, there was no significant difference in perceived friend support between boys and girls.

### 3.2. Results for the Models

This study used three models to examine the mediating effect of perceived friend support. The first model used gender role attitudes to predict the mediating variable—perceived friend support. The second model used gender role attitudes to predict bullying behaviors. In the third model, gender role attitudes and perceived friend support were used to predict bullying behaviors.

Models 3-1 to 3-3 show the binary logistic regression and ordinal logistic regression results for the male sample. The results of model 3-1 indicate that after controlling for other variables, there was a significant positive association between gender role attitudes and perceived friend support (adjusted OR = 1.055; SE = 0.013). That is, the greater the value of gender role attitudes, the more egalitarian the attitude was and the more support the individual perceived from friends. Model 3-2 suggests that there was a significant association between gender role attitudes and bullying. Specifically, the more equal the gender role attitude, the less likely the individual was to bully others (adjusted OR = 0.974; SE = 0.013). Therefore, Hypothesis 1 was supported. In model 3-3, the mediating variable of perceived friend support was added, and results suggest that perceived friend support influenced bullying behaviors. Compared to those who perceived more friend support, adolescent males who perceived less friend support were more likely to bully others (adjusted OR = 2.082; SE = 0.392). The results indicate that: first, the direct effect of gender role attitudes was marginally significant (significant at 0.1 level); and second, gender role attitudes indirectly affected bullying behaviors through perceived friend support. That is, perceived friend support mediated the relationship between gender role attitudes and bullying behaviors. Such findings support Hypothesis 2.

In addition, results of the Sobel–Goodman Test suggest that perceived friend support did mediate the association between gender attitudes and bullying among adolescent boys. Specifically, 18.6% of gender attitudes’ effect on bullying behaviors was mediated by perceived friend support.

Further, the present study found that compared with those who rated their family economic status as better, boys with average and worse family economic status perceived less support from friends (adjusted OR = 0.610, SE = 0.089; adjusted OR = 0.477, SE = 0.094). Also, boys from urban areas perceived more support from friends than those from rural areas (adjusted OR = 1.529, SE = 0.193). For adolescent boys, academic achievement was a predictor of bullying behaviors: adolescent boys who had passed all their exams in the previous semester were less likely to bully than those who had failed (adjusted OR = 0.732, SE = 0.086; adjusted OR = 0.722, SE = 0.085). For parental problem behaviors, the more problem behaviors the father and mother had, the more likely adolescent boys were to bully (adjusted OR = 1.606, SE = 0.181; adjusted OR = 1.594, SE = 0.180).

## 4. Discussion

The present study is one of the few attempts to investigate the relationship between gender role attitudes, perceived friend support, and school bullying among male adolescents in China. Specifically, our findings demonstrated that traditional gender role attitudes are the driving force for bullying behaviors among male adolescents: The more traditional their gender role attitudes are, the more likely they will be the perpetrators of school bullying. Additionally, our findings confirmed the mediating role of perceived friend support, meaning that male adolescents with more traditional gender role attitudes will report lower levels of perceived friend support, which leads to higher risk of bullying perpetration.

Consistent with previous literature [40,42,46,47,48], the present investigation also found that male adolescents hold more conservative gender beliefs than females. Moreover, one of the most important contributions of this study is to link traditional gender role attitudes with bullying perpetration among male students. For male adolescents, gender traditionality means a greater identification with normative masculine beliefs that encourage toughness, self-reliance, social dominance, and aggressiveness [51] and higher likelihood of acting out by bullying to prove themselves.

Additionally, the study highlights the importance of using the masculinity theory in understanding bullying among male adolescents. Existing studies have concentrated on associations between hegemonic masculinity and gender-based violence, including partner violence [52,55,56], dating violence [57,58,59,60,61], and sexual violence and harassment [43,61], etc. The present investigation hence contributes to the current research by providing empirical evidence for associations between traditional masculinity and bullying. For male adolescents, on one hand, bullying could be a strategy for them to gain power and attention among peers and establish their masculinity. On the other hand, hegemonic masculinity may restrain male adolescents from expressing themselves and seeking help, which in turn reduces friend support [36].

Our findings also support the stress-buffering theory. In particular, male adolescents who perceive insufficient friend support may fail to deal with stress and therefore adopting bullying as their coping strategy. This study provides empirical evidence that friend support mediates the relationship between gender role attitudes and bullying behaviors. More traditional gender role attitudes restrain or reduce the peer support available to individuals, which increases the likelihood of bullying behaviors.

## 5. Limitations

The present study bears several limitations. First, it employed a cross-sectional method, and hence the relationships between variables are correlational, rather than causal. This means that we are unable to make causal inferences based on the present results. For a clearer examination of the relationships, longitudinal designs are advised for future studies.

Second, the study was conducted among 11 high schools in two cities in China, which cannot be generalized to all adolescent boys in China. Nationwide randomized sampling methods should be adopted for better generalizability of findings.

Third, although masculinity theories are used as a gendered mechanism linking gender role attitudes and bullying, the study did not measure traditional masculinity directly. Therefore, future studies examining relationships between traditional masculinity and bullying should adopt more accurate assessments of masculinity endorsement.

Last, this study examined only the effect of gender role attitudes and perceived friend support on bullying behaviors and focused on the role of perpetrators within school bullying. However, given the complex dynamics in school bullying, roles of victims and bystanders should also be included in future research.

## 6. Practical Implications

This empirical study has echoed the calls of the Sustainable Development Goals, especially SDG 4, which aim to establish inclusive and equitable quality education and “provide safe, non-violent, inclusive and effective learning environments for all” [83]. Findings of the study show that to achieve the goal, a number of things need to be done. In China, sexuality education is often stigmatized. However, given present findings on the direction of association between gender role attitudes and bullying, our study shows that promoting gender equality education and school bullying prevention may be a strategic approach to conduct comprehensive sexuality education. Accordingly, for a more inclusive and equitable school climate, schools are advised to enact zero-tolerance anti-bullying policies and advocate for positive, mutually respectful, and supportive peer relationships.

Further, it is urgent to battle hegemonic masculinity by promoting healthy masculinity among male adolescents. Such efforts require collaboration between schools and families. For parents, alternative ways of boys’ socialization are needed. In particular, kindness and tenderness, as well as emotional expression and help-seeking, should be encouraged. For schools, bystander intervention training that facilitates refusal to engage in bullying and helping behaviors of victims are needed. Collectively, these efforts may prevent boys from engaging in bullying and gender-based violence when they grow older.

## 7. Conclusions

Our study indicates that promoting healthy masculinity can be a good entry point to conduct interventions on male adolescent bullying. The present study explored the impact of rigid gender role attitudes on adolescent boys’ bullying behaviors. Furthermore, the study confirms that friend support is one of the pathways through which masculinity influences bullying among male adolescents. In 2018, UNESCO published the International Technical Guidance on Sexuality Education (revised version) [84]. Compared with the earlier version, the new guidance incorporated two key concepts: understanding gender (key concept 3) and violence and staying safe (key concept 4). This study is a great example to integrate the two new key concepts with the traditional concept of relationships (key concept 1). Collectively, this study delivers a clear message to male adolescents that they need to abandon toxic masculinity, improve life and communication skills, establish healthy friendships, and prevent themselves from involvement in school bullying and other types of violence.

## Figures and Tables

**Table 1:** Description of sociodemographic characteristics of the male adolescent sample (*n* = 1624).

	*n* (%) or Mean (SD)
Age	14.94 (1.282)
Educational stage	
Junior high school (%)	729 (44.89)
Senior high school (%)	895 (55.11)
Household register	
Rural (%)	614 (37.81)
Urban (%)	1010 (62.19)
Only child or not	
Yes (%)	573 (35.28)
No (%)	1051 (64.72)
Pass exam or not	
Yes (%)	569 (35.04)
No (%)	1055 (64.96)
Education of father (years)	11.761 (3.640)
Education of mother (years)	10.751 (4.122)
Parents’ marital status	
Married (%)	1437 (88.49)
Other (%)	187 (11.51)
Family economy	
Better (%)	272 (16.75)
Average (%)	1119 (68.90)
Worse (%)	233 (14.35)
Fathers’ problem behaviors	0.660 (0.977)
Mothers’ problem behaviors	0.172 (0.525)

Categorical variables are described by frequency (percentage) and continuous variables are described by mean (standard deviation). Range of age is 12–20.

**Table 2:** Description of bullying perpetration, gender role attitudes, and perceived friend support for male (1624) and female (1548) adolescents.

	All	Male	Female	T-Test or Chi-Square Test	
	*n* (%) or Mean (SD)	*n* (%) or Mean (SD)	*n* (%) or Mean (SD)	*t* Value/Chi Squared	*p*
Bullying				8.25	***
Yes	1068 (33.67)	585 (36.02)	483 (31.20)		
No	2104 (66.33)	1039 (63.98)	106 (68.80)		
Gender role attitude	30.471 (0.082)	28.314 (0.104)	32.733 (0.098)	30.78	***
Perceived friend support				1.19	n.s.
Low	667 (21.03)	339 (20.87)	328 (21.19)		
Medium	2105 (66.36)	1070 (65.89)	1035 (66.86)		
High	400 (12.61)	215 (13.24)	185 (11.95)		

Range of gender role attitudes is 1–40. *** = *p* < 0.001. n.s. = *p* > 0.05. *p* refers to the tests for gender difference.

**Table 3:** Binary logistic regression and ordinal logistic regression predicting bullying and friend support for male students (*n* = 1624).

	Model 3-1		Model 3-2		Model 3-3	
	Perceived Friend Support		Bullying		Bullying	
	OR	SE	OR	SE	OR	SE
Gender role attitudes	1.055 ***	0.013	0.974 *	0.013	0.979	0.013
Perceived friend support (high = 0)						
Medium					1.137	0.159
Low					2.082 ***	0.392
Age	0.955	0.062	0.889	0.059	0.885	0.059
Educational stages (senior high school = 0)	0.801	0.136	0.944	0.162	0.911	0.158
Household register (rural = 0)	1.529 **	0.193	0.937	0.120	0.968	0.125
Only child or not(no = 0)	0.958	0.111	1.021	0.123	1.020	0.124
Pass exam or not(no = 0)	1.077	0.121	0.732 **	0.086	0.722 **	0.085
Parents’ marital status (other = 0)	0.966	0.159	1.105	0.188	1.099	0.188
Fathers’ years of education	0.994	0.019	1.017	0.020	1.014	0.020
Mothers’ years of education	1.000	0.016	0.990	0.017	0.990	0.017
Family economy(better = 0)						
Average	0.610 **	0.089	0.777	0.116	0.754	0.114
Worse	0.477 ***	0.094	0.958	0.189	0.888	0.177
Fathers’ problem behaviors	0.957	0.067	1.318 ***	0.077	1.308	0.078
Mothers’ problem behaviors	0.834	0.088	1.606 ***	0.181	1.594 ***	0.180
Pseudo R squared	0.0255		0.0399		0.0482	

Note * = *p* < 0.05; ** = *p* < 0.01; *** = *p* < 0.001. Dummy variables representing the schools are also included in the models.

## Data Availability

The data are not publicly available due to privacy and ethical concerns.

## References

[1] UNESCO Let’s Decide How to Measure School Violence. https://unesdoc.unesco.org/ark:/48223/pf0000246984.

[2] Gladden R.M., Vivolo-Kantor A.M., Hamburger M.E., Lumpkin C.D. Bullying Surveillance among Youths: Uniform Definitions for Public Health and Recommonded Data Elements, Version 1.0. https://www.cdc.gov/violenceprevention/pdf/bullying-definitions-final-a.pdf.

[3] Olweus D. (1993). Bullying at School: What We Know and What We Can Do.

[4] UNESCO School Violence and Bullying: Global Status Report. https://healtheducationresources.unesco.org/library/documents/school-violence-and-bullying-global-status-and-trends-drivers-and-consequences.

[5] UNESCO Behind the Numbers: Ending School Violence and Bullying. https://unesdoc.unesco.org/ark:/48223/pf0000366483.

[6] UNICEF Ending the Torment: Tackling Bullying from the Schoolyard to Cyberspace. https://sustainabledevelopment.un.org/content/documents/2577tackling_bullying_from_schoolyard_to_cyberspace_low_res_fa.pdf#page=136.

[7] Chan H.C.O., Wong D.S.W. (2015). Traditional School Bullying and Cyberbullying in Chinese Societies: Prevalence and a Review of the Whole-School Intervention Approach. Aggress. Violent Behav..

[8] Xue J., Hu R., Chai L., Han Z., Sun I.Y. (2022). Examining the Prevalence and Risk Factors of School Bullying Perpetration among Chinese Children and Adolescents. Front. Psychol..

[9] Shen Y., Xin T., Zhang J., Lin H. (2019). School Bullying in Four Regions in China and Intervention Strategies: Evidence from PISA 2018. Educ. Policy Rev. China.

[10] Zhang Q. (2020). The Performance Analysis and Future Prospect of Chinese Anti-Bullying Policy and Practice: Evidence from PISA 2015 and PISA 2018. Explor. Educ. Dev..

[11] OECD PISA 2015: Results in Focus. https://www.oecd.org/pisa/pisa-2015-results-in-focus.pdf.

[12] Schleicher A. PISA 2018: Insights and Interpretations. https://www.oecd.org/pisa/PISA%202018%20Insights%20and%20Interpretations%20FINAL%20PDF.pdf.

[13] Nansel T.R., Overpeck M., Pilla R.S., Ruan W.J., Simons-Morton B., Scheidt P. (2001). Bullying Behaviors among U.S. Youth. JAMA.

[14] Chang F., Lee C., Chui C., Hsi W., Huang T., Pan Y. (2013). Relationships among Cyberbullying, School Bullying, and Mental Health in Taiwanese Adolescents. J. Sch. Health.

[15] Kozasa S., Oiji A., Kiyota A., Sawa T., Kim S.Y. (2017). Relationship between the Experience of Being a Bully/Victim and Mental Health in Preadolescence and Adolescence: A Cross-Sectional Study. Ann. Gen. Psychiatry.

[16] Hysing M., Askeland K.G., La Greca A.M., Solberg M.E., Breivik K., Sivertsen B. (2021). Bullying Involvement in Adolescence: Implications for Sleep, Mental Health, and Academic Outcomes. J. Interpers. Violence.

[17] Guo J., Li M., Wang X., Ma S., Ma J. (2020). Being Bullied and Depressive Symptoms in Chinese High School Students: The Role of Social Support. Psychiatry Res..

[18] Luo X., Zheng R., Xiao P., Xie X., Liu Q., Zhu K., Wu X., Xiang Z., Song R. (2022). Relationship between School Bullying and Mental Health Status of Adolescent Students in China: A Nationwide Cross-Sectional Study. Asian J. Psychiatr..

[19] Inchley J., Currie D., Budisavljevic S., Torsheim T., Jåstad A., Cosma A., Kelly C., Arnarsson Á.M. Spotlight on Adolescent Health and Well-Being: Findings from the 2017/2018 Health Behaviour in School-Aged Children (HBSC) Survey in Europe and Canada: International Report, Volume 1. Key Findings. https://apps.who.int/iris/bitstream/handle/10665/332091/9789289055000-eng.pdf.

[20] UN Protecting Children from Bullying: Report of the Secretary-General. https://violenceagainstchildren.un.org/content/protecting-children-bullying-report-secretary-general.

[21] Chen J.K., Chen L.M. (2020). A Cross-National Examination of School Violence and Nonattendance Due to School Violence in Taiwan, Hong Kong, and Mainland China: A Rasch Model Approach. J. Sch. Violence.

[22] Teng H., Yao J. (2018). A Study on Influencing Factors on School Bullying and Primary and Secondary Education. Educ. Sci. Res..

[23] Wang H., Wang Y., Wang G., Wilson A., Jin T., Zhu L., Yu R., Wang S., Yin W., Song H. (2021). Structural Family Factors and Bullying at School: A Large Scale Investigation Based on a Chinese Adolescent Sample. BMC Public Health.

[24] Wang X., Liu L., Chen B., Chui W.H., Wang X. (2022). The Mediating Effect of Self-Control on the Relationship between Exposure to Violence and Fighting among Chinese Secondary School Students. J. Sch. Violence.

[25] Davis S.N., Greenstein T.N. (2009). Gender Ideology: Components, Predictors, and Consequences. Annu. Rev. Sociol..

[26] Eagly A.H., Wood W., Van Lange P.A., Kruglanski A.W., Higgins E.T. (2012). Social Role Theory. Handbook of Theories of Social Psychology.

[27] Steinfeldt J.A., Vaughan E.L., LaFollette J.R., Steinfeldt M.C. (2012). Bullying among Adolescent Football Players: Role of Masculinity and Moral Atmosphere. Psychol. Men Masc..

[28] Gereš N., Orpinas P., Rodin U., Štimac-Grbić D., Mujkić A. (2021). Bullying and Attitudes toward Masculinity in Croatian Schools: Behavioral and Emotional Characteristics of Students Who Bully Others. J. Interpers. Violence.

[29] Carrera-Fernández M.V., Lameiras-Fernández M., Rodríguez-Castro Y., Vallejo-Medina P. (2013). Bullying among Spanish Secondary Education Students: The Role of Gender Traits, Sexism, and Homophobia. J. Interpers. Violence.

[30] Malecki C.K., Demaray M.K. (2002). Measuring Perceived Social Support: Development of the Child and Adolescent Social Support Scale (CASSS). Psychol. Sch..

[31] Collins W.A., Laursen B. (2004). Changing Relationships, Changing Youth: Interpersonal Contexts of Adolescent Development. J. Early Adolesc..

[32] Bokhorst C.L., Sumter S.R., Westenberg P.M. (2010). Social Support from Parents, Friends, Classmates, and Teachers in Children and Adolescents Aged 9 to 18 Years: Who Is Perceived as Most Supportive?. Soc. Dev..

[33] Heerde J.A., Hemphill S.A. (2018). Examination of Associations between Informal Help-Seeking Behavior, Social Support, and Adolescent Psychosocial Outcomes: A Meta-Analysis. Dev. Rev..

[34] Šmigelskas K., Vaičiūnas T., Lukoševičiūtė J., Malinowska-Cieślik M., Melkumova M., Movsesyan E., Zaborskis A. (2018). Sufficient Social Support as a Possible Preventive Factor against Fighting and Bullying in School Children. Int. J. Environ. Res. Public Health.

[35] Kendrick K., Jutengren G., Stattin H. (2012). The Protective Role of Supportive Friends against Bullying Perpetration and Victimization. J. Adolesc..

[36] McKenzie S.K., Collings S., Jenkin G., River J. (2018). Masculinity, Social Connectedness, and Mental Health: Men’s Diverse Patterns of Practice. Am. J. Mens. Health.

[37] Zhang J., Zuo X., Yu C., Lian Q., Tu X., Lou C. (2021). The Association between Gender Role Attitudes and Alcohol Use among Early Adolescents in Shanghai, China. Subst. Use Misuse.

[38] Taga F., Kimmel M., Hearn J., Connel R.W. (2005). East Asian Masculinities. Handbook of Studies on Men and Masculinities.

[39] Li Z., Yang H., Zhu X., Xie L. (2021). A Multilevel Study of the Impact of Egalitarian Attitudes toward Gender Roles on Fertility Desires in China. Popul. Res. Policy Rev..

[40] Sa Z., Tian L., Wang X. (2021). Evidence for a Comprehensive Sexuality Education Intervention That Enhances Chinese Adolescents’ Sexual Knowledge and Gender Awareness and Empowers Young Women. Sex Roles.

[41] Shi Y. (2009). A Study on the Development of Gender Role Attitudes in Middle School Students and Its Relationship with Parent Gender-Related Cognition. Master’s Thesis.

[42] Xi H., Zuo X., Yu C., Lian Q., Tu X., Yan L., Lou C. (2020). Gender Stereotypes of Early Adolescents and Its Influencing Factors. Chin. J. Sch. Health.

[43] Zuo X., Lou C., Gao E., Lian Q., Shah I.H. (2018). Gender Role Attitudes, Awareness and Experiences of Non-Consensual Sex among University Students in Shanghai, China. Reprod. Health.

[44] Wu H., Luo S., Espinosa-Hernández G., Klettner A., White T.D., Li H. (2019). Relating Gender to Sex: Gendered Attitudes, Sexual Double Standard, Sexual Intentions and Behaviors in Two Chinese Adolescent Samples. J. Sex Res..

[45] Su Y., Cui C., Xu D. (2020). Gender Egalitarianism of Children and Youth and Its Impacting Factors. Ment. Health Educ. Prim. Second. Sch..

[46] Halimi M., Consuegra E., Struyven K., Engels N. (2016). The Relationship between Youngsters’ Gender Role Attitudes and Individual, Home, and School Characteristics: A Review. SAGE Open.

[47] Zuo X., Lian Q., Cheng Y., Tu X., Wang Z., Yu C., Lou C. (2013). Gender Role Development and Gender Stereotype of Adolescents in China. Chin. J. Hum. Sex..

[48] Tang W., Gai X., Zhao Y. (2011). A Survey on Children and Youth’s Gender Egalitarianism. J. Inn. Mong. Norm. Univ..

[49] Connell R.W. (2005). Masculinities.

[50] Connell R.W., Messerschmidt J.W. (2005). Hegemonic Masculinity: Rethinking the Concept. Gend. Soc..

[51] Leone R.M., Parrott D.J., Ireland J.L., Birch P., Ireland C.A. (2018). Hegemonic Masculinity and Aggression. The Routledge International Handbook of Human Aggression.

[52] Fulu E., Warner X., Miedema S., Jewkes R., Roselli T., Lang J. Why Do Some Men Use Violence against Women and How Can We Prevent It?. Summary Report of Quantitative Findings from the United Nations Multi-Country Study on Men and Violence in Asia and the Pacific..

[53] Jewkes R., Jordaan E., Myrttinen H., Gibbs A. (2020). Masculinities and Violence: Using Latent Class Analysis to Investigate the Origins and Correlates of Differences between Men in the Cross-Sectional UN Multi-Country Study on Men and Violence in Asia and the Pacific. J. Glob. Health.

[54] Wang X., Fang G., Li H. A Study on Gender-Based Violence and Masculinity in China: The Quantitative Report. https://china.unfpa.org/sites/default/files/pub-pdf/6.Research%20on%20Gender-based%20Violence%20and%20Masculinities%20in%20China_Quantitativ.pdf.

[55] Wang X., Fang G., Li H. (2019). Gender-Based Violence and Hegemonic Masculinity in China: An Analysis Based on the Quantitative Research. China Popul. Dev. Stud..

[56] Santana M.C., Raj A., Decker M.R., La Marche A., Silverman J.G. (2006). Masculine Gender Roles Associated with Increased Sexual Risk and Intimate Partner Violence Perpetration among Young Adult Men. J. Urban Health.

[57] Reyes H.L.M.N., Foshee V.A., Niolon P.H., Reidy D.E., Hall J.E. (2016). Gender Role Attitudes and Male Adolescent Dating Violence Perpetration: Normative Beliefs as Moderators. J. Youth Adolesc..

[58] Shen A.C.T., Chiu M.Y.L., Gao J. (2012). Predictors of Dating Violence among Chinese Adolescents: The Role of Gender-Role Beliefs and Justification of Violence. J. Interpers. Violence.

[59] Reed L.A., Ward L.M., Tolman R.M., Lippman J.R., Seabrook R.C. (2021). The Association between Stereotypical Gender and Dating Beliefs and Digital Dating Abuse Perpetration in Adolescent Dating Relationships. J. Interpers. Violence.

[60] Miller E., Culyba A.J., Paglisotti T., Massof M., Gao Q., Ports K.A., Kato-Wallace J., Pulerwitz J., Espelage D.L., Abebe K.Z. (2020). Male Adolescents’ Gender Attitudes and Violence: Implications for Youth Violence Prevention. Am. J. Prev. Med..

[61] Reidy D.E., Smith-Darden J.P., Cortina K.S., Kernsmith R.M., Kernsmith P.D. (2015). Masculine Discrepancy Stress, Teen Dating Violence, and Sexual Violence Perpetration among Adolescent Boys. J. Adolesc. Health.

[62] Birkett M., Espelage D.L. (2015). Homophobic Name-Calling, Peer-Groups, and Masculinity: The Socialization of Homophobic Behavior in Adolescents. Soc. Dev..

[63] Poteat V.P., Kimmel M.S., Wilchins R. (2011). The Moderating Effects of Support for Violence Beliefs on Masculine Norms, Aggression, and Homophobic Behavior during Adolescence. J. Res. Adolesc..

[64] Valido A., Merrin G.J., Espelage D.L., Robinson L.E., Nickodem K., Ingram K.M., El Sheikh A.J., Torgal C., Fairclough J. (2021). Social-Ecological Predictors of Homophobic Name-Calling Perpetration and Victimization among Early Adolescents. J. Early Adolesc..

[65] David D.S., Brannon R. (1976). The Forty-Nine Percent Majority: The Male Sex Role.

[66] Warren L.W. (1983). Male Intolerance of Depression: A Review with Implications for Psychotherapy. Clin. Psychol. Rev..

[67] Mahalik J.R., Lagan H.D., Morrison J.A. (2006). Health Behaviors and Masculinity in Kenyan and U.S. Male College Students. Psychol. Men Masc..

[68] Wester S.R., Christianson H.F., Vogel D.L., Wei M. (2007). Gender Role Conflict and Psychological Distress: The Role of Social Support. Psychol. Men Masc..

[69] Exner-Cortens D., Wright A., Claussen C., Truscott E. (2021). A Systematic Review of Adolescent Masculinities and Associations with Internalizing Behavior Problems and Social Support. Am. J. Community Psychol..

[70] Cohen S., Wills T.A. (1985). Stress, Social Support, and the Buffering Hypothesis. Psychol. Bull..

[71] Chui W.H., Chan H.C.O. (2013). Association between Self-Control and School Bullying Behaviors among Macanese Adolescents. Child Abus. Negl..

[72] Espelage D.L., Holt M.K. (2001). Bullying and Victimization during Early Adolescence: Peer Influences and Psychosocial Correlates. J. Emot. Abus..

[73] Wen Y., Zhu X., Haegele J.A., Yu F. (2022). Mental Health, Bullying, and Victimization among Chinese Adolescents. Children.

[74] Galambos N.L., Petersen A.C., Richards M., Gitelson I.B. (1985). The Attitudes toward Women Scale for Adolescents (AWSA): A Study of Reliability and Validity. Sex Roles.

[75] Zimet G.D., Dahlem N.W., Zimet S.G., Farley G.K. (1988). The Multidimensional Scale of Perceived Social Support. J. Pers. Assess..

[76] Chen S. (2019). Chinese Adolescents’ Emotional Intelligence, Perceived Social Support, and Resilience-The Impact of School Type Selection. Front. Psychol..

[77] Miao H., Sun H., He X., Zhang Z., Nie Q., Guo C. (2021). Perceived Social Support and Life Satisfaction among Young Chinese Adolescents: The Mediating Effect of Psychological Suzhi and Its Components. Curr. Psychol..

[78] Erginoz E., Alikasifoglu M., Ercan O., Uysal O., Alp Z., Ocak S., Oktay Tanyildiz G., Ekici B., Yucel I.K., Albayrak Kaymak D. (2015). The Role of Parental, School, and Peer Factors in Adolescent Bullying Involvement: Results from the Turkish HBSC 2005/2006 Study. Asia-Pacific J. Public Health.

[79] Thomas H.J., Connor J.P., Scott J.G. (2018). Why Do Children and Adolescents Bully Their Peers? A Critical Review of Key Theoretical Frameworks. Soc. Psychiatry Psychiatr. Epidemiol..

[80] Radliff K.M., Wheaton J.E., Robinson K., Morris J. (2012). Illuminating the Relationship between Bullying and Substance Use among Middle and High School Youth. Addict. Behav..

[81] Xu S., Ren J., Li F., Wang L., Wang S. (2020). School Bullying among Vocational School Students in China: Prevalence and Associations with Personal, Relational, and School Factors. J. Interpers. Violence.

[82] Hong J.S., Espelage D.L. (2012). A Review of Research on Bullying and Peer Victimization in School: An Ecological System Analysis. Aggress. Violent Behav..

[83] UN Do You Know All 17 SDGs?. https://sdgs.un.org/goals.

[84] UNESCO International Technical Guidance on Sexuality Education. https://www.unfpa.org/sites/default/files/pub-pdf/ITGSE.pdf.

